# Evolving evidence implicates cytomegalovirus as a promoter of malignant glioma pathogenesis

**DOI:** 10.1186/2042-4280-2-10

**Published:** 2011-10-26

**Authors:** Charles S Cobbs

**Affiliations:** 1 California Pacific Medical Center Research Institute, 475 Brannan Street, San Francisco, CA, 94114, USA; 2 University of California, San Francisco, Department of Neurological Surgery, 505 Parnassus Avenue, San Francisco, CA, 94143, USA

## Abstract

Human cytomegalovirus (HCMV) was first reported to be strongly associated with human malignant gliomas in 2002. HCMV is a herpesvirus that causes congenital brain infection and multi-organ disease in immumocompromised individuals. Malignant gliomas are the most common and aggressive adult brain tumors and glioblastoma multiforme (GBM), the highest grade glioma, is associated with a life expectancy of less than two years. HCMV gene products encode for multiple proteins that can promote the various signaling pathways critical to tumor growth, including those involved in mitogenesis, mutagenesis, apoptosis, inflammation, angiogenesis, invasion and immuno-evasion. Several groups have now demonstrated that human malignant gliomas are universally infected with HCMV and express gene products that can promote key signaling pathways in glioma pathogenesis. In this review I discuss specific HCMV gene products that we and others have recently found to be expressed in GBM *in vivo*, including the HCMV IE1, US28, gB and IL-10 proteins. The roles these HCMV gene products play in dysregulating key pathways in glioma biology, including the PDGFR, AKT, STAT3, and monocyte/microglia function are discussed. Finally, I review emerging human clinical trials for GBM based on anti-HCMV strategies.

## Glioblastoma Clinical

Malignant gliomas are the most common cerebral tumors, account for about 4% of cancer deaths, and grade IV gliomas, glioblastoma multiforme (GBM), are the most aggressive [1]. Despite recent advances in radiotherapy and chemotherapy, the prognosis for GBM patients is a median survival after diagnosis of about 14 months [1]. No significant gains in the understanding of the etiology of GBM has occurred in the last several decades, and except for very rare genetic cancer syndromes and exposure to ionizing radiation, there is no known cause for gliomas [2].

## Glioma biology

Despite the lack of a known etiological agent for GBM, recent findings have led to important insights into molecular pathways involved in gliomagenesis. These findings suggest that GBM cells may arise from two pathways. One potential pathway would involve post-mitotic astrocytic cells that have de-differentiated into immature tumor cells. Alternatively, the tumor arises by the immortalization and transformation of resident neuo-glial precursor stem cells (NPCs) in the adult brain [3,4].

One of the most important recent observations regarding the biology of GBM is that these tumors, like other hematological and solid tumors, are comprised of a subpopulation of tumor initiating cells with stem-like characteristics, often termed "cancer stem cells" or glioma stem cells (GSC) [5,6]. These cells often express the CD133 surface glycoprotein, and are therefore defined as CD133+ cells [5,7]. GSCs likely represent the most important targets of the GBM cells in terms of therapy, since these cells are more resistant to radiation and chemotherapy, and also may be immunosuppressive [7-9]. These cells also appear to play a critical role in re-initiating tumor growth after standard therapies, and thus may play a central role in tumor recurrence. Indeed, in vitro, these cells can act as tumor-founding cells down to the single cell level, and the tumors they produce in animal models closely resemble the main histological properties of GBM [10].

Key signaling pathways involved in promoting GBM pathogenesis in the susceptible cell types. ie., NPCs, are those that lead to sustained activation of receptor tyrosine kinase (RTK) signaling pathways such as EGFR and PDGFRα, and downstream PI3-K/AKT pathways, as well as those that inactivate important tumor suppressor pathways such as p16(*INK4a*), Rb, p53 and PTEN [3,11,12]. For example, sustained activation of PDGFRα in susceptible neuroglial precursor stem cells within the adult brain may promote the earliest steps of gliomagenesis. In adult mice, infusion of PDGF into the environment of NPCs that express PDGFRα can lead to early hyperpastic astrocytic lesions that have many characteristics of early stage gliomas [13].

An increased understanding of the role of stem cell maintenance factors in sustaining GSCs has led to the increased appreciation that key regulators of self-renewal, such as Sox2 and Bmi-1 may be important for sustaining viability of the GSC population [14-16]. Another important transcriptional activator that controls GSC maintenance and is also important in myriad other signaling pathways in GBM cells that control angiogenesis, tumor cell invasion, apoptosis and immunosuppression, is signal transducer and activator of transcription 3 (STAT3) [8,9,17-19]. Indeed, STAT3 transcriptional activation, along with activation of CEBPb, is thought to play a fundamental role in the transition of GBM cells into those types of GBMs with the most aggressive, mesenchymal, features and poor survival [20].

## HCMV

Ten years ago, our group became interested in the possibility that a viral infection might be involved in the pathogenesis of GBM since our research indicated that these tumors had characteristics of a chronic inflammatory state [21,22]. Human cytomegalovirus (HCMV) was a potential candidate since HCMV is the most common infection of human fetal brain, and HCMV was known to be able to persistently infect glioma cells and become reactivated in a chronic inflammatory state and in the setting of immunosuppression [23-26]. In 2002, we reported that HCMV proteins and nucleic acids could be detected in virtually all GBMs evaluated, but not in normal brain or other benign brain tumors [27]. Although these findings were not reproduced initially [28,29], several groups were subsequently able to confirm these findings using highly sensitive techniques we developed that detect low levels of viral expression [30-34].

## History of HCMV in cancer

Although in 2002 HCMV was not considered to be an oncogenic virus, previous work by multiple investigators had suggested that HCMV could promote oncogenesis. Initially, Rapp and colleagues in the 1970s had demonstrated that urogenital isolates of HCMV could transform cells [35]. Subsequently in the 1980's and 1990's, other groups determined that HCMV possessed potentially oncogenic gene products [36,37]. A group led by Cinatl et al demonstrated multiple oncomodulatory aspects of HCMV over the ensuing years (reviewed in [38,39]). The current thinking among experts in this area is that HCMV does not possess acute transforming activity; rather HCMV gene products may be "oncomodulatory". In such a situation, expression of HCMV gene products in an established tumor may accelerate progression of the tumor by influencing multiple key pathways such as angiogenesis, invasion, mitogenesis, immunomodulation, etc.

## Mechanisms of glioma promotion

Upon discovering that HCMV infection was prevalent in malignant gliomas, we attempted to determine whether viral infection might promote glioma pathogenesis. Since the HCMV IE1 gene product was readily detected in tumors, and since this viral gene was previously known to be mutagenic and a potent viral transcriptional activator [38,40], we sought to determine the impact of IE1 expression in GBM cells. We found that IE1 expression caused increased proliferation of GBM cell lines and primary explant cells [41]. This phenomenon was associated with IE1-mediated inactivation of the p53 and Rb tumor suppressor proteins, and activation of the PI3-K/AKT signaling pathway [41]. IE1 promoted cell cycle entry and DNA synthesis of human glioma cells on both stable expression in tumor-derived cell lines as well as transient expression in primary glioblastoma cells. Our findings were consistent with those of others who have demonstrated that the HCMV IE1 gene product can block p53 transcriptional activity and induce a dominant negative p53 family member protein [42,43].

These findings were also consistent with those from another group demonstrating that IE1 expression in a GBM cell line could lead to decreased expression of thrombospondin-1 (TSP-1), GFAP, and p53 [44]. Since TSP-1 inhibits angiogenesis and GFAP is associated with a more differentiated astrocytic phenotype [45], these results suggested that, in addition to promoting glioma cell mitogenesis, expression of IE1 in glioma cells may also promote GBM angiogenesis, and a de-differentiated state, along with loss of tumor suppressor activity. Interestingly, we found that expression of HCMV IE1 in normal human astrocytes or normal fibroblasts resulted in either no change in proliferation or a decreased proliferation, respectively. This result suggests that expression of HCMV IE1 may have a paradoxical effect on cells based on their neoplastic or differentiation state - promoting proliferation in neoplastic cells while exerting the opposite effect on wild-type cells.

In the last few years, other groups have made further advances with respect to elucidating how HCMV gene products may impact gliomagenesis. In 2009, Straat et al. showed that IE1 expression in GBM cells was associated with induction of telomerase activity [32]. HCMV IE1 protein stimulated hTERT promoter activity, and in specimens of GBM, HCMV IE and hTERT proteins were co-localized in malignant cells and their levels paralleled each other. Since telomerase activation can lead to cellular immortalization and telomerase activation has been observed in 90% of cancers [46], these findings suggest that HCMV IE1 mediated activation of telomerase may contribute to oncogenesis in glioma.

Consistent with these observations, Scheurer et al. found that the level of IE1 expression in malignant gliomas was positively correlated to the grade of tumor, with GBM having the highest levels of expression [31]. In addition, the level of IE1 expression in GBM has been found to inversely correlate with patient survival [47].

While IE1 expression in a tumor cell may promote important oncogenic signaling pathways, we also became interested in the possibility that HCMV might promote sustained RTK activation, which is a hallmark of GBM pathogenesis. We and others had observed that HCMV attachment caused activation of RTK signaling, and that a RTK was potentially causing HCMV-mediated PI3-K/AKT signaling [48]. In exploring this phenomenon, we discovered that activation of the PDGFRα receptor was essential for HCMV infection [49]. Furthermore, we observed that the HCMV gB envelope glycoprotein binds specifically to PDGFRα upon viral attachment and functions like the authentic ligand PDGF in terms of activating downstream RTK signaling of the PI3-K/AKT signaling pathway [49]. Blockade of PDGFRα with a blocking antibody or with the PDGFRα small molecule inhibitor Gleevec^® ^completely inhibited HCMV entry into the cell, viral expression and replication [49]. These findings immediately raised the possibility that expression of the HCMV gB glycoprotein on the cell surface of glial precursor stem cells or GBM cells that overexpress PDGFRα may facilitate autocrine or paracrine activation of the PDGFRα signaling pathway, which plays a major role in glioma pathogenesis. Ongoing studies in our laboratory are investigating this hypothesis.

In addition to sustained mitogenesis and inhibition of tumor suppressor function, another key event in oncogenesis is blockade of cellular differentiation. If HCMV infection of NPCs could promote the PDGFRα - PI3K/AKT signaling pathway while simultaneously blocking their ability to differentiate, this would greatly increase the likelihood of neoplastic transformation. This hypothetical scenario could occur in HCMV infected NPCs, since these cells are fully permissive to HCMV infection [50] and since HCMV gene products block the ability of NPCs to differentiate into neurons and astrocytes [51-53].

## HCMV and the US28 - STAT3 Signaling Pathway

Since the STAT3 signaling pathway may play a major role as a master regulator of glioma pathogenesis, HCMV gene products that promote STAT3 transcriptional activation could influence glioma biology. One such HCMV gene product is the US28 chemokine receptor, which is *bona fide *viral oncoprotein [54]. US28 binds a broad spectrum of chemokines, including SDF-1, CCL2/MCP-1, CCL5/RANTES, and CX3CL1/fractalkine, and, unlike its human cellular homolog CCR1, US28 exhibits constitutive activity [55]. Ectopic expression of US28 induces a pro-angiogenic, transformed glioma phenotype *in vivo*, by upregulating VEGF [54]. The induction of VEGF expression as a result of HCMV infection in U373 GBM cells was due to the constitutive activation of US28, since a US28-deficient mutant HCMV did not induce VEGF [54]. Thus, the angiogenic (i.e., aggressive) phenotype in some GBMs that express US28 might be due to the oncogenic properties of US28 acting in concert with other viral proteins, such as the IE1 and IE2 gene products, to facilitate tumor initiation and progression after infection. Consistent with these data, our preliminary microarray and TaqMan analyses indicate that both IE1 and US28 are highly expressed in patient-derived GBM biopsy specimens. In addition to its ability to induce oncogenic transformation and promote angiogenesis, US28 promotes cell migration toward chemokines RANTES and MCP-1,[56] which are abundantly expressed in malignant gliomas [57].

Recently, Slinger et al. showed that increased concentrations of VEGF and IL-6 are secreted in supernatants of US28-expressing cells and that this resulted in downstream activation of STAT3 [58]. They determined that STAT3 is essential for the US28-mediated proliferative phenotype described above. In GBM specimens from patients, they found that US28 co-localized with pSTAT3 in the vascular niche of the tumor and that US28 induces proliferation in HCMV-infected tumors by establishing a positive feedback loop through activation of the IL-6-STAT3 signaling axis [58]. These data strongly implicate the HCMV US28 gene product as a major driver of STAT3 signaling in GBM, a role that would implicate US28 in GBM angiogenesis, invasion, and immune evasion.

In addition, to explore the potential role of HCMV US28 in colon cancer, Bongers et al. recently described a transgenic mouse that expresses US28 in intestinal epithelial stem cells [59]. Strikingly, these mice developed colon adenomas and adenocarcinomas by 40 weeks of age, a phenomenon that was enhanced by the presence of US28 stimulatory cytokine CCL2. They noted that the Wnt signaling pathway was activated in the US28+ tumor cells as demonstrated by US28-mediated phosphorylation (inactivation) of GSK3-β, and subsequent dephosphorylation (activation) of β-catenin, and induction of downstream Wnt target genes survivin, cyclin-D1, and *c-*myc [59]. In summary, both of these recent reports indicate that HCMV US28 can drive oncogenic signaling through two key pathways that are involved in cancer stem cell maintenance and glioma proliferation and invasion--STAT3 and GSK3-β/β-catenin.

## Immune Evasion

A critical component in inflammation-associated malignancies like gliomas is the loss of normal antitumor immune function in the tumor microenvironment. In addition to the tumor-promoting effects of HCMV infection of monocytes/macrophages, expression of HCMV gene products by GBM cells could dramatically alter the host's immune response to tumor. A variety of tumor-derived factors contribute to the emergence of complex local and regional immunosuppressive networks, including VEGF, IL-10, TGF-β, and PGE-2 [60,61]. Cytotoxic T lymphocyte (CTL; CD8+) and NK cell responses are critical effectors of normal host antitumor immunosurveillance. Through millions of years of co-evolution with the host, HCMV has evolved multiple strategies to allow persistent viral infection through a complex array of immune evasion strategies [62-66]. Several HCMV gene products are expressed as immediate early and early viral genes to block the host-cell MHC class I antigen expression, which is required for CD8+ cytotoxic tumor killing. The UL83 gene product pp65, which our laboratory and others have consistently detected in GBM cells, blocks antigen presentation of IE1, one of the earliest immunodominant HCMV epitopes, from CD8+ T cells.

Through a complex interaction with tumor cells and tumor associated microglia/macrophages (TAMs), HCMV infection is also likely to impair function of tumor antigen presentation by dendritic cells (DCs) in the tumor microenvironment. IL-10 suppresses the maturation and cytokine production of DCs, key regulators of adaptive immunity, and prevents the activation and polarization of naive T cells toward protective IFN-γ-producing effectors. Treatment of immature DCs with supernatant from HCMV-infected cultures has been found to inhibit both the lipopolysaccharide-induced DC maturation and pro-inflammatory cytokine production [67]. Not surprisingly, use of IL-10 is a common mechanism for intracellular pathogens to suppress or delay the immune response and establish productive infection in the host [68].

To maximize this strategy, HCMV encodes an UL111A gene product that has 27% identity with human IL-10 (cmvIL-10) and that has potent immunosuppressive properties. cmvIL-10 inhibits mononuclear cell proliferation, suppresses inflammatory cytokine production, and downregulates MHC expression [69]. cmvIL-10 alters the earliest host responses to viral antigens by dampening the magnitude and specificity of innate effector cells [70]. In addition, there is a commensurate reduction in the quality and quantity of early and long-term, HCMV-specific adaptive immune responses [70]. Further studies show that cmvIL-10 inhibits DC maturation and migration [71], effects that are likely to significantly hamper the cell-mediated immune response to HCMV infection. Since cmvIL-10 is likely expressed during early stages of HCMV infection of glioma cells, this would potentially provide these HCMV-positive tumor cells an unheralded survival advantage against host innate and adaptive immune effector cells.

Recently, Dzurisinsky et al., used flow cytometry to analyze GBMs for evidence of HCMV antigens [72]. They found that GSCs are preferentially infected *in vivo *by HCMV. They also evaluated tumors for evidence of cmvIL-10 production by ELISA and found that HCMV showed a tropism for GSCs and macrophages/microglia within GBMs. Furthermore, these tumor GSCs produced cmvIL-10, which induced human monocytes to assume an M2 immunosuppressive phenotype (as manifested by downmodulation of the major histocompatibility complex and costimulatory molecules) while upregulating immunoinhibitory B7-H1. The cmvIL-10-treated monocytes produced angiogenic VEGF, immunosuppressive TGF-beta, and enhanced migration of GSCs. Thus, their findings indicate that HCMV triggers a feedforward mechanism of gliomagenesis *in vivo *by inducing tumor-supportive monocytes.

## Animal model

A recently described model of murine CMV (MCMV) infection in mice predisposed to spontaneously arising GBMs (Mut3 mice) indicates that CMV infection can promote gliomagenesis and GBM pathogenesis. To test this hypothesis, Price et al. infected Mut3 (*GFAP-cre*; *Nf1*loxP/+; *Trp53*-/+) mice with MCMV [73]. Mut3 mice develop normally, but eventually succumb from malignant astrocytomas, including GBM, at adult age with almost complete penetrance. MCMV infection significantly shortened survival of Mut3 mice with increased incidence of GBM compared to anaplastic astrocytomas (WHO grade III). Before tumor formation, there was a significant increase in the Gfap- or nestin-positive NPC population by MCMV infection. Mice infected with MCMV contained an abnormal area of increased cellularity in the subventricular zone near the midline bilaterally. The cells had spindle-shaped nuclei and condensed chromatin. Interestingly, this is the area where NPCs reside. However, there were no noticeable changes in S100b-positive pan glia or NeuN-positive neurons. These data suggest that CMV infection accelerates GBM progression by affecting the NPC population.

## Therapeutic implications

The observations described above led a research team at the Karolinska Institute in Stockholm to initiate a clinical trial of the antiviral drug Valcyte in glioblastoma patients. This Phase II prospective randomized clinical trial began in 2006 via an investigational grant from Roche to investigators Söderberg-Naucler, Peredo and Stragliotto http://clinicaltrials.gov/ct2/show/study/NCT00400322. Using a randomized, double blind design to test the safety and efficacy of the drug, 42 patients were enrolled. Patients received 900 mg Valcyte twice daily for three weeks followed by a maintenance dose (900 mg once daily) for an additional 21 weeks. MRI scans were performed pre and post operatively, and at 12 and 24 weeks. No serious adverse events were clearly linked to Valcyte treatment. Subject followup is still ongoing.

Work by Duke University scientists has resulted in an immunotherapy approach to attack CMV infection in GBM. Clinical results to date also support the concept that an anti-CMV approach to this disease may have clinical benefit. A Phase I/II immunotherapy clinical trial of autologous CMV pp65 RNA loaded dendritic cells (DCs) was initiated in 2006 (ATTAC Protocol- FDA-IND-BB-12839; Duke IRB Protocol 8108; PI: Duane A. Mitchell). This trial enrolled 13 patients with newly diagnosed GBM who underwent gross total resection (> 95%) followed by standard external beam radiation (60 Gy) and concurrent temozolomide (TMZ) (75 mg/m^2^/d) for six weeks followed by monthly 5 day TMZ (150-200 mg/m^2^/d) for six cycles. Leukapheresis harvested post surgical resection and prior to initiation of TMZ was used to generate DCs and pp65 RNA electroporated autologous DCs (2 × 10^7 ^DCs i.d.) were administered every two weeks for the first three doses after first TMZ cycle and monthly thereafter on day 21 of each cycle. Patients were monitored by MRI (every two months) for tumor progression and blood was collected monthly for immunologic monitoring. Initial results are highly encouraging. Patients exhibited a median progression-free survival (PFS) of 15.4 months and an overall survival OS of 20.6 months. Both outcomes are highly significant compared to matched historical controls (p = 0.004). Duke investigators have plans to pursue this immunotherapy strategy with a second generation peptide based vaccine to CMV to be delivered alongside with lymphopenia inducing doses of TMZ to GBM patients. This "PEP-CMV" Phase I/II clinical trial will be pursued at multiple institutions through the NCI Brain Tumor SPORE mechanism.

## Summary

**Figure 1:**
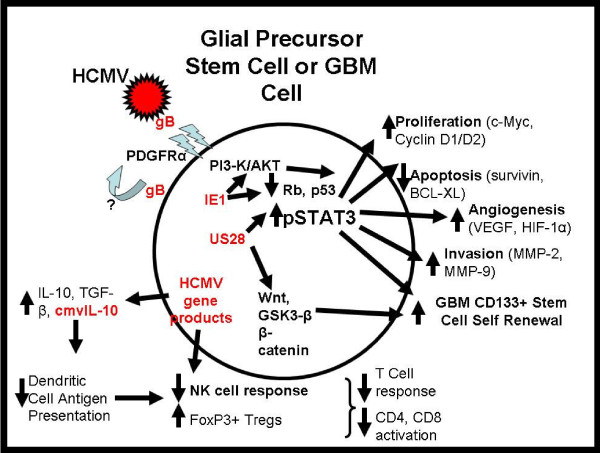
**HCMV promotes GBM pathogenesis**. HCMV utilizes multiple mechanisms to promote oncogenesis and subvert the host anti-tumor immune function. HCMV envelope glycoprotein B (gB) attaches to and activates PDGFRα signaling. HCMV gene products IE1 and US28 drive multiple cellular pathways important in gliomagenesis such as PI3-K/AKT, pSTAT3, and GSK3-β. The STAT3 pathway is a master regulator of glioma proliferation, apoptosis, angiogenesis, invasion and tumor stem cell maintenance. Other HCMV gene products, and the cmvIL-10 cytokine, lead to further expression of host factors like IL-10 and TGFβ which subvert host anti-tumor immune responses.

Growing evidence indicates that HCMV infection occurs in malignant gliomas in vivo and that HCMV gene products can promote important oncogenic pathways and phenotypes that likely contribute to glioma pathogenesis. The implications of this growing field are that HCMV infection is not merely an epiphenomenon in the glioma neoplastic process; rather viral gene expression can promote tumor aggressiveness and possibly play a causal role in gliomagenesis. Observations to date implicate the HCMV IE1, gB, IL-10 and US28 gene products as tumor promoters in gliomagenesis (Figure 1). Levels of HCMV gene products are correlated with glioma grade and patient survival. An animal model of CMV infection in the setting of gliomagenesis suggests that CMV can promote the progression of developing gliomas to becoming GBMs, possibly by enhanced mitogenesis within the vulnerable NPC population. Preliminary clinical trials in humans with GBM suggest that both direct antiviral therapeutic interventions and vaccine based therapies may impact tumor progression and increase patient survival. This cumulative body of data supports further investigations into the role of HCMV in malignant glioma pathogenesis and therapy.

## Competing interests Statement

The author declares that they have no competing interests.
